# Next-Generation Sequencing Enables Spatiotemporal Resolution of Human Centromere Replication Timing

**DOI:** 10.3390/genes10040269

**Published:** 2019-04-02

**Authors:** Dashiell J. Massey, Dongsung Kim, Kayla E. Brooks, Marcus B. Smolka, Amnon Koren

**Affiliations:** 1 Department of Molecular Biology and Genetics, Cornell University, Ithaca, NY 14853, USA; dm792@cornell.edu (D.J.M); dk662@cornell.edu (D.K.); keb253@cornell.edu (K.E.B.); 2 Weill Institute for Cell and Molecular Biology, Cornell University, Ithaca, NY 14853, USA

**Keywords:** DNA replication timing, centromeres, heterochromatin, next-generation sequencing

## Abstract

Centromeres serve a critical function in preserving genome integrity across sequential cell divisions, by mediating symmetric chromosome segregation. The repetitive, heterochromatic nature of centromeres is thought to be inhibitory to DNA replication, but has also led to their underrepresentation in human reference genome assemblies. Consequently, centromeres have been excluded from genomic replication timing analyses, leaving their time of replication unresolved. However, the most recent human reference genome, hg38, included models of centromere sequences. To establish the experimental requirements for achieving replication timing profiles for centromeres, we sequenced G_1_- and S-phase cells from five human cell lines, and aligned the sequence reads to hg38. We were able to infer DNA replication timing profiles for the centromeres in each of the five cell lines, which showed that centromere replication occurs in mid-to-late S phase. Furthermore, we found that replication timing was more variable between cell lines in the centromere regions than expected, given the distribution of variation in replication timing genome-wide. These results suggest the potential of these, and future, sequence models to enable high-resolution studies of replication in centromeres and other heterochromatic regions.

## 1. Introduction

DNA replication during the S phase of the cell cycle initiates at replication origin loci, which are both spatially dispersed across the genome and asynchronously activated. The resultant spatiotemporal pattern of DNA replication timing is highly reproducible and largely conserved, producing consistent early- and late-replicating regions (reviewed in [1]). In general, early-replicating regions show greater transcriptional activity [2,3,4,5 and higher gene density [2,3,4,5,6], while late-replicating regions tend to accumulate more mutations [7,8,9,10]. Constitutive heterochromatin is widely accepted as a prime example of the relationship between closed chromatin state and late replication timing [11,12,13,14]. Late replication of heterochromatic regions has been linked to telomeric proximity [15,16] and transcriptional silencing [16,17] in the budding yeast *Saccharomyces cerevisiae,* and to histone hypoacetylation [18] and distance from the nuclear periphery [19] in mouse.

Centromeres are an intriguing potential outlier to the late-replicating heterochromatin paradigm: centromeres replicate early in multiple yeast species, including *S. cerevisiae* [20], the fission yeast *Schizosaccharomyces pombe* [21,22], and the pathogenic yeast *Candida albicans* [23]. This presents an opportunity for insight into the mechanisms that promote replication origin activity as well as the mechanisms that dictate late replication in other heterochromatic regions. Indeed, in *S. pombe*, early centromeric replication has been explained by interactions of heterochromatin protein 1 (HP1) with the replication initiation factors CDC6 [24] and DDK [25]. Ablation of either of these interactions results in the centromere replicating late with other heterochromatin [24,25], thus giving further support to the model that a closed chromatin state is generally repressive to origin firing.

The time at which centromeres replicate is less clear in higher eukaryotes: centromere replication in early S phase has been reported in the *Drosophila Kc* cell line [26], in mid-S phase in multiple human cell lines [27], in late S phase in *Drosophila* larvae [28], and even throughout the full S phase in mouse cell lines [29]. However, the general consensus is that human centromeres replicate late in S phase [30,31,32], consistent with the timing of heterochromatin replication. Studies across species have also suggested that centromeres on different chromosomes may replicate at different times [28,29,30,32] and that the neighboring pericentromeric heterochromatin replicates earlier than the centromeres themselves [29,32].

Next-generation sequencing provides a high-resolution assay for replication timing at genome-scale [8,33]. However, in most eukaryotes, centromeres are satellite-rich constitutive heterochromatic regions ranging in size from hundreds of kilobases to several megabases. The high repetitive-sequence content of centromeres renders them difficult to sequence and assemble. As a result, centromeres have historically been gaps in reference genomes [34] and thus excluded from newer, sequencing-based analyses. However, the most recent human reference genome, hg38, includes sequence models for all 24 centromeres [35]. These constructed sequences take advantage of subtle variation within related centromeric satellites to build localized assemblies that are then arranged by a second-order Markov chain modeled on the frequency of these variants [34]. Although the sequence models do not necessarily reflect the accurate linear DNA sequence within the centromere, they do allow sequencing reads originating from the centromeres to be aligned.

Here, we report that the centromere sequence models in hg38 enable measuring replication timing of human centromeres. We reveal their timing and variation in five cell lines, and detail the experimental conditions required to obtain this type of information. Our results demonstrate that high-throughput sequencing of human cell lines is both a feasible and a fruitful methodology to clarify a more detailed understanding of the human centromere and its time of replication during the S phase of the cell cycle.

## 2. Materials and Methods

### 2.1. Tissue Culture

HEK293T and A2780 cells were cultured in Dulbecco’s modified Eagle medium (Corning Life Sciences, Tewksbury, MA, USA) supplemented with 10% fetal bovine serum (FBS; Corning). GM12878, HCC1143, and HCC1954 were grown in Roswell Park Memorial Institute 1640 medium (Corning) supplemented with 15% FBS. All cell lines were obtained from the American Type Culture Collection or the Coriell Institute, and grown at 37 °C in a 5% CO_2_ atmosphere.

### 2.2. Fluorescence-Activated Cell Sorting

Asynchronous populations of ~50 million cells were fixed in 70% ethanol, treated with RNase A (10 mg/mL) for 30 min at 37 °C, and stained with propidium iodide (1 mg/mL) in the dark for 30 min at room temperature. Stained cells were flow-sorted on a FACSAria II (BD Biosciences, San Jose, CA, USA) to isolate 1 million G_1_- and 1 million S-phase cells.

### 2.3. Library Preparation and Sequencing

DNA was isolated using the MasterPure™ DNA Purification Kit (Epicentre, Madison, WI, USA) and libraries were prepared with the TruSeq DNA PCR-Free Library Prep Kit (Illumina, Inc., San Diego, CA, USA). Paired-end sequencing was performed for 75 cycles with the Illumina NextSeq 500 (A2780 and HEK293T; Cornell University Biotechnology Resource Center, Ithaca, NY) or for 150 cycles with the Illumina HiSeq X Ten (GM12878, HCC1143, and HCC1954; GENEWIZ, Inc., South Plainfield, NJ, USA).

### 2.4. Sequence Alignment

Sequence reads were aligned to the human reference genome hg38 using the Burrows–Wheeler Aligner maximal exact matches (BWA-MEM) algorithm (bwa v0.7.13). For HEK293T and A2780, quality-filtered reads were combined from two independent genomic libraries (HEK293T) or two independent sequencing runs (A2780) to enhance read depth. Centromere coordinates were obtained from the UCSC Genome Browser (University of California, Santa Cruz), genome build GRCh38/hg38. To account for repetitive sequences that might be represented as single copies in the reference genome (thus inflating estimates of copy number), reads were binned in 100 Kb windows, and the 99th percentile of windows with the highest read coverage were excluded. Similarly, regions with low mappability were filtered by binning reads in 500 Kb windows and excluding the bottom 0.5% lowest coverage windows. Ploidy was estimated using GenomeSTRiP [36].

### 2.5. Replication Timing Profiles

Replication timing profiles were generated as in [8]. Briefly, the G_1_-phase cells were used to define sliding chromosome windows of equal read depth (200 reads), which were then used to bin the reads from the S-phase cells. Outlier read depth values were filtered using a piecewise segmentation model (MATLAB function segment, with assumed variance 0.04). Contiguously mapped segments between gaps in the reference genome were smoothed with a cubic smoothing spline (MATLAB function csaps, smoothing parameter 1 × 10^−16^). Data were then normalized to an autosomal mean of 0 and standard deviation of 1.

### 2.6. Data Availability

Sequence data reported in this study have been submitted to the Sequence Read Archive (SRA) under accession number PRJNA419407. Smoothed replication timing profiles are available at http://amnonkoren.com/data.

## 3. Results

### 3.1. Genome-Wide Replication Timing Profiles for Five Human Cell Lines

To assess the feasibility of studying centromere replication by whole-genome sequencing, we generated replication timing profiles for five human cell lines: an apparently healthy lymphoblastoid cell line (GM12878; [37]), an embryonic kidney cell line (HEK293T), an ovarian carcinoma cell line (A2780), and two breast cancer cell lines (HCC1143 and HCC1194; [38]). For each cell line, an asynchronous population was flow-sorted to isolate 1 million cells from the G_1_ (pre-replicative) and S (replicative) phases of the cell cycle. Pairs of G_1_- and S-phase fractions were sequenced and aligned to hg38.

For each cell line, replication timing was inferred for the S-phase fraction in variable-size windows determined by the G_1_-phase fraction (Figure S1A), as previously described [8]. Briefly, early-replicating regions are expected to be overrepresented (i.e., have high sequencing read depth) in the S-phase cell fraction, while late-replicating regions will be underrepresented. The G_1_-phase cell fraction, for which all genomic regions are expected to be present in uniform copy number, was used as a baseline to account for mappability and sequencing biases, as well as copy-number variants (Figure S2).

To assess the quality of these replication timing profiles, we considered the autocorrelation of replication timing as a function of genomic distance. Consistent with the spatiotemporal dynamics of replication, each profile demonstrated high autocorrelation along the chromosome on the scale of several megabases (Figure S1B). Autosome-wide replication timing profiles were strongly correlated across samples (r = 0.70–0.85) and with our previously-published measurements in lymphoblastoid cell lines [8] (r = 0.75–0.96; Figure S1C), consistent with previous reports that at least 50% of the replication timing is conserved between cell types [33,39].

### 3.2. Replication Timing Can Be Profiled in Centromeric Regions by Paired-End Sequencing

**Figure 1:**
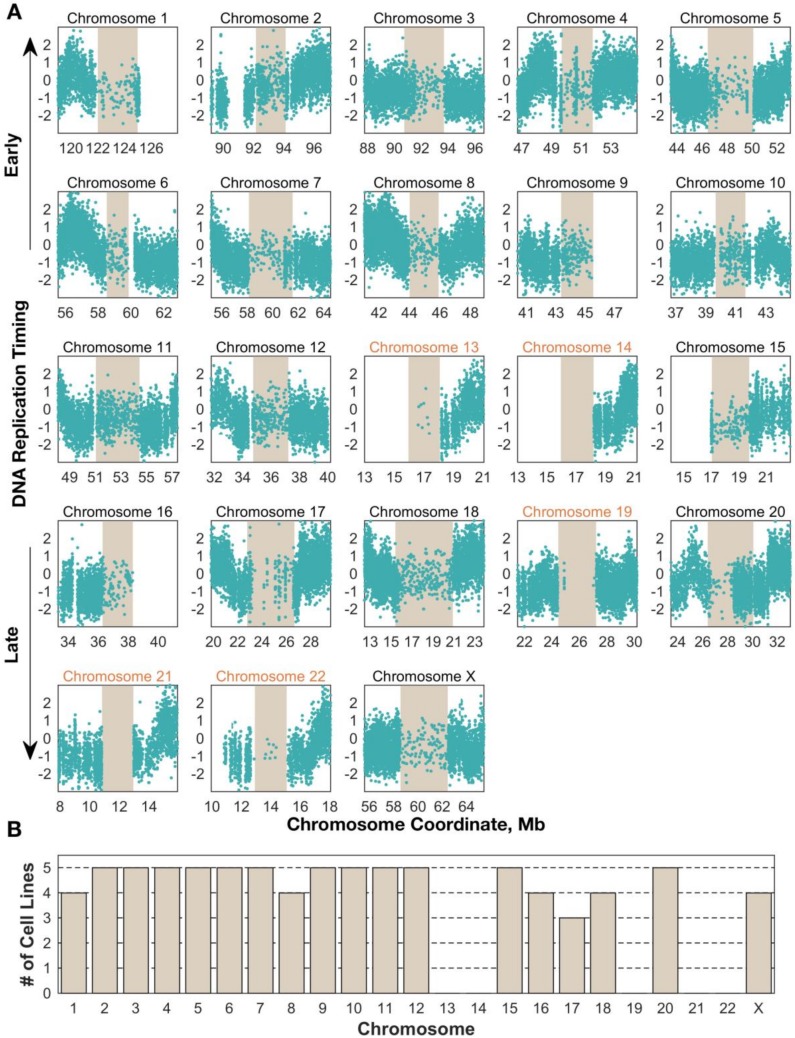
Centromere replication timing can be consistently measured in human cell lines for most chromosomes. (**A**) Unsmoothed replication timing data for the breast cancer cell line HCC1954 across all centromeres (tan) and flanking regions. Each dot represents a single window, defined by 200 reads in the G_1_ phase sample. Chromosomes labeled in black contain at least 10 centromeric windows and were included in the analyses for Figure 3, Figure 4, Figure 5 (**B**) Replication timing inference is successful in the same subset of centromeres across cell lines. Bars represent the number of cell lines in which that chromosome’s centromere contained enough windows to be included in further analyses.

We next focused our attention on the centromeres, requiring that any centromere contain on average at least 10 G_1_-defined windows (1100 reads) per megabase to be included in our analysis. For A2780, 13 centromeres were successfully profiled, while 17–18 centromeres passed this threshold in the other four cell lines (Figure 1A). The identity of the centromeres that were successfully profiled was consistent across samples, suggesting that this is a property of the sequence models of individual centromeres (Figure 1B).

**Figure 2:**
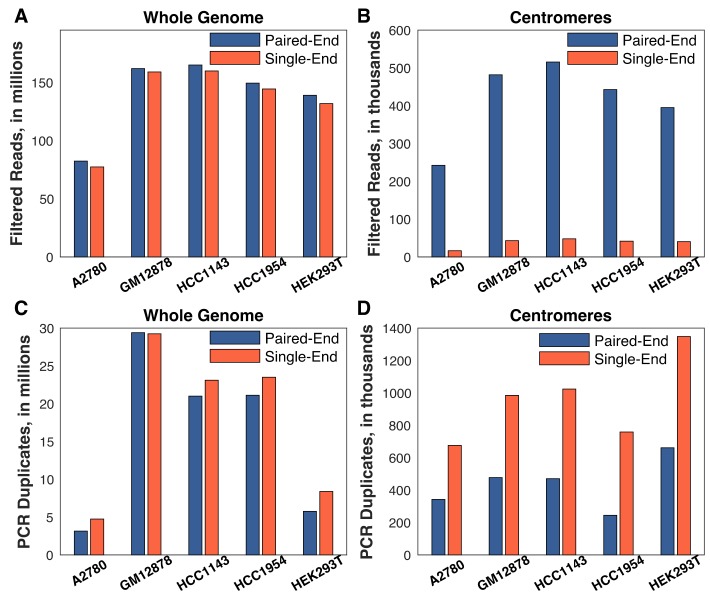
Paired-end sequencing is critical for obtaining centromere replication timing. Single-end sequencing was generated by considering only the first read of each pair. Read (or read-pair) counts were averaged across the G_1_ and S phase fractions for each cell line. (**A**,**B**) Single-end alignment had a negligible effect on read depth genome-wide, but eliminated almost all of the reads in the centromeres. (**C**,**D**) The difference between single- and paired-end sequencing is largely driven by the difficulty in discriminating true sequence repeats from PCR and optical duplicates with single-end reads. All chromosomes/centromeres were considered for this analysis.

The smaller number of successful centromere profiles in A2780 prompted us to consider the effect of sequencing read depth on the ability to infer replication timing profiles: this cell line was sequenced to approximately half the coverage of the others (~80 million filtered read pairs vs. ~140–165 million in the others; Figure 2A, blue bars). We hypothesized that because centromeres are highly repetitive, reads derived from those regions would be disproportionately likely to be flagged as poorly-mapped or as PCR duplicates during alignment. Indeed, a large proportion (~85%) of centromeric reads for all samples were flagged as “poorly mapped” and excluded (Figure S3).

Strikingly, while total read depth was an important factor in obtaining sufficient usable centromere sequence reads, paired-end sequencing proved to be even more crucial for the success of our approach. We re-aligned the sequencing data, considering only the first read of each read pair, and found that there was a negligible difference genome-wide in the number of reads passing quality filtering when using single-end sequencing (Figure 2A, red bars). In contrast, there was a roughly ten-fold reduction in the number of centromeric reads passing quality filtering (Figure 2B, red bars). In addition, there was a disproportionate loss of reads in A2780: there was on average a 2.6-fold difference in the number of single-end centromeric reads in A2780 (16,367 reads) relative to the other cell lines (HEK293T: 40,408 reads; HCC1954: 41,585; GM12878: 42,854 reads; HCC1143: 47,861 reads). The importance of paired-end sequencing was largely due to the ability to discriminate technical repeats from true repetitive centromeric sequences (Figure 2C,D). With single-end reads, identical reads are likely to be falsely flagged as PCR or optical duplicates. Paired-end sequencing ameliorates this issue because the probability of observing a non-unique read-pair is much lower than the probability of a non-unique single read. Together, these results establish the technical requirements for mapping DNA replication timing in human centromeres: an order of 100 million or more total reads, and, most importantly, paired-end sequencing.

### 3.3. Centromere Replication Occurs in Mid-to-Late S Phase and Varies among Cell Lines

**Figure 3:**
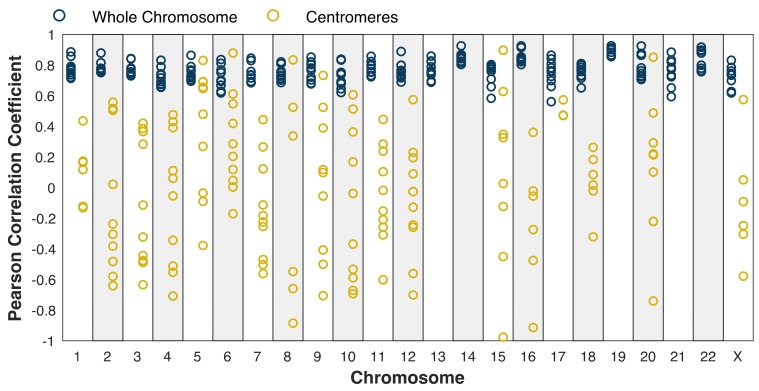
Centromere replication timing is more variable between cell lines than chromosome-wide replication timing. Blue: whole chromosome (excluding centromere); gold: centromeres. Pearson correlation was calculated for each mappable centromere (see Figure 1) and for each chromosome for each pair of cell lines. Dots represent individual pairwise comparisons. The difference in the distribution of correlation coefficients between the centromeres and whole chromosomes is robust when controlling for the size of the centromeres (Figure S5).

Given the ability to measure centromeric replication timing, we next compared these profiles among the five cell lines. We first noted a relatively large variability in centromere replication timing among cell lines. The centromere of a given chromosome replicated earlier than the genome average in some cell lines, but later than the genome average in other cell lines (Figure S4). While chromosome-wide correlations were relatively high (r = 0.49–0.92; Figure 3, blue dots), the correlations within the centromeres were much less consistent, ranging from r = −0.98 to r = 0.89 (Figure 3, gold dots). Some centromeres, for instance, on chromosome 5, appeared to be more similar across samples (r > 0.5 in six pairwise comparisons). In contrast, other centromeres, such as on chromosome 8, were highly similar between some pairs (r = 0.83) but highly dissimilar between others (r = −0.86). This broad distribution of correlation coefficients was significantly different than would be expected for random genomic regions, controlling for the size of the centromeres (Figure S5A) and for the small number of centromeric windows relative to genomic windows (Figure S5B).

**Figure 4:**
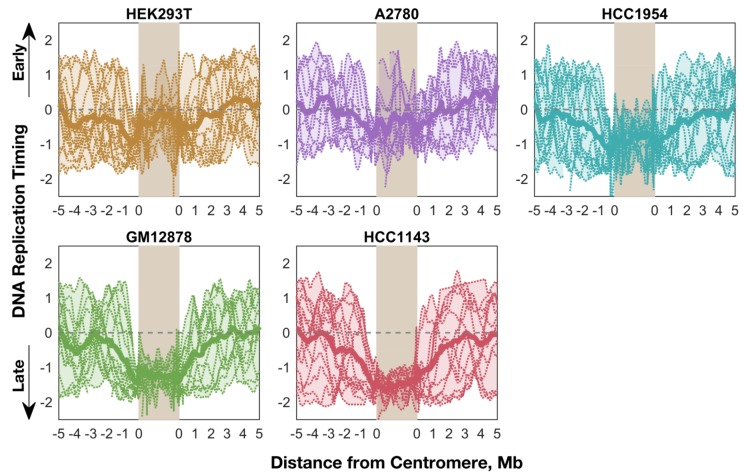
Average replication timing is more consistent within the centromeres of a given cell line than in the surrounding pericentromeres (or the whole genome). For each cell line, the replication timing profile for each mappable centromere (see Figure 1) is shown, overlaid with an averaged “aggregate” profile for that cell line’s centromeres. The shaded area indicates the minimum and maximum values, and the dashed line indicates the genome average. Each centromere was divided into 100 bins for the purpose of aggregation.

We next analyzed the pattern and timing of centromere replication by aggregating centromeres across all chromosomes within each cell line. Although the centromeres of individual chromosomes did not display consistent replication timing across cell lines, centromeres within a given cell line were notably similar, particularly in the cell lines with later average centromere replication (Figure 4). This trend did not extend to the pericentromeric regions (or genome-wide), which showed much more variable replication timing values within individual cell lines. This observation potentially points to suppression of centromere replication in these cell lines, or alternatively less stringent control of centromeric replication timing in other cell lines.

**Figure 5:**
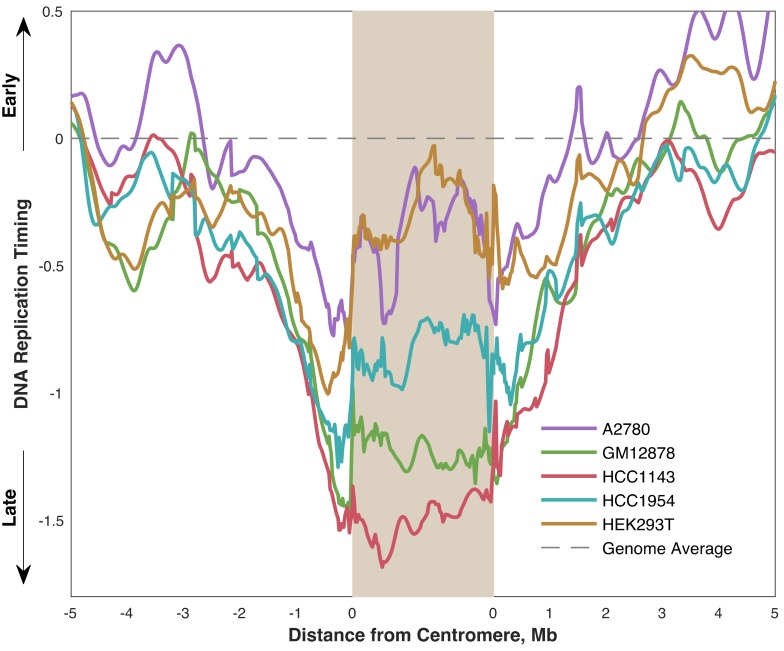
Centromere replication timing is variable between cell lines, occurring between mid- and mid-late S phase. Each line represents the average replication timing of mappable centromeres (see Figure 1) in the indicated cell line.

We also found that the pericentromeric regions replicated progressively later towards the centromeres, indicating that centromeres are embedded within a local area of relatively late replication (Figure 4). This was even more noticeable when overlaying the aggregate profiles to compare centromeric replication between cell lines (Figure 5). However, within the centromeres themselves, this trend either plateaued (GM12878 and HCC1143), or even seemed to reverse (A2780, HCC1954, and HEK293T) such that the centromeres were not later-replicating (or were even earlier-replicating) than their surrounding pericentromeres. Strikingly, the centromeres in A2780 and HEK293T appeared to replicate very close to the genome average, while the other three cell lines showed centromeric replication in mid-to-late S phase. Based on these data, we suggest that human centromeres are not late-replicating, but instead replicate close to mid-S phase and often earlier than their surrounding pericentromeric regions.

## 4. Discussion

How and when human centromeres replicate remains an open question. While most studies in mammalian systems have concluded that centromeres replicate in mid-to-late S phase [30,31,32], several previous reports from human and mouse cell lines have also found evidence that centromeres replicate earlier than other heterochromatic regions [27,29,32,40]. In addition, human centromeres are transcriptionally active [41], which is generally associated with early replication [2,3,4,5]. Thus, despite the well-accepted notion that heterochromatin replicates late in S phase, centromeres appear to comprise a specialized type of chromatin with its own unique biology.

Using the latest human reference genome, hg38, we find that centromeres replicate in mid-to-late S phase, while the neighboring heterochromatin replicates markedly later, in agreement with previous reports that the centromeres replicate earlier than their surroundings [26,29,32]. Intriguingly, two of the cell lines in our study—A2780 and HEK293T—replicated their centromeres close to the genome average, i.e., firmly in the middle of S phase.

We measure DNA replication timing without cell synchronization or fractioning S phase, capturing S phase as a continuous process. In addition, this is the first study to our knowledge to assay DNA replication timing of mammalian centromeres without nucleotide analogue incorporation. These methodological advancements enable us to generate high-resolution replication timing data for centromeres in the context of the whole genome. Furthermore, we are able to assay almost all of the centromeres in a chromosome-specific way, rather than using antibodies against centromeric histones [29], centromere-specific probes [27,30,31] or a pan-centromere consensus probe [32]. These advantages allow us to observe that centromere replication timing was more variable between the studied cell lines than other regions of the genome. Inter-chromosome variability in centromere replication has previously been reported [28,29,30,32] but these studies have lacked the resolution to ascribe that variation to particular chromosomes.

The apparently early replication timing of the centromeres relative to the surrounding pericentromere is compatible with evidence from previous reports that centromeres contain active replication origins. Molecular combing has suggested that replication initiation sites are observed at the same density in centromeric regions compared to other genomic regions, and that α-satellite monomers bind the origin recognition complex in vitro—both of which imply the existence of active replication origins within centromeres [32]. These origins likely interact with specialized chromatin modifiers to promote origin firing within a generally repressive chromatin context. However, because the centromere reference models we used were assembled probabilistically and not from linear sequencing reads through the centromere, we cannot be confident that the centromere-wide sequence is in the correct order. Thus, at present, the centromere sequence models are not sufficient to generate contiguous replication profiles, from which the locations of these replication origins could be predicted.

We demonstrate here for the first time that human centromeric replication timing can be inferred by high-throughput sequencing, and establish the technical requirements and the importance of paired-end sequencing for assaying centromere replication. As newer linear centromere reference sequences become available [42], this approach will prove to be valuable in identifying the specific locations of centromeric replication origins and characterizing variation among cell lines. In establishing a straightforward method for detecting changes in replication timing of centromeres, we open the door to genetic assays which will help to better characterize the chromatin modifiers that are important for replication activity within these heterochromatin domains.

## Supplementary Materials

The following are available online at https://www.mdpi.com/2073-4425/10/4/269/s1, Figure S1: Replication timing profiles of five human cell lines, Figure S2: Copy number in 1Mb windows for the G1-phase fractions, following mappability- and GC-bias correction using GenomeSTRiP, Figure S3: Approximately 85% of read pairs mapped to centromeres are flagged as low-quality and removed prior to analysis, Figure S4: Cell lines display variation in centromeric replication timing across all chromosomes, Figure S5: The broad distribution of pairwise correlations for centromeric regions is significantly different than expected by chance.
