# Supplementary material for: Next-Generation Sequencing Enables Spatiotemporal Resolution of Human Centromere Replication Timing
Source: Genes (Basel). 2019 Apr 2;10(4):269. doi: 10.3390/genes10040269 (PMC6523654; doi:10.3390/genes10040269)
Supplement: Supplementary file 1 [file genes-10-00269-s001.pdf]

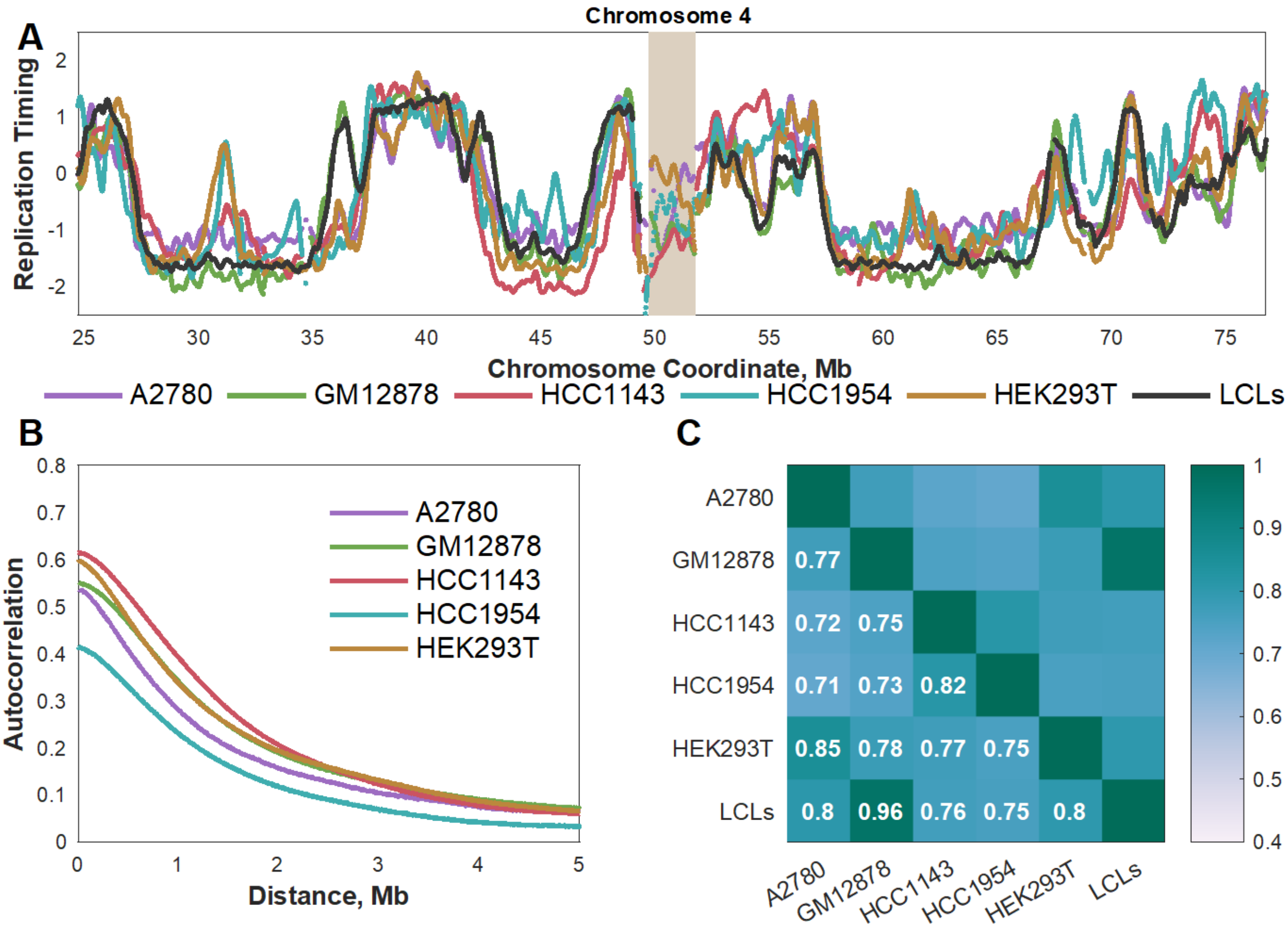

Figure S1. Replication timing profiles of five human cell lines. (A) Replication timing for a region of chromosome 4, centered on the centromere (shaded). All samples were compared to an average replication timing profile of six lymphoblastoid cell lines (labelled "LCLs", from [1]). **(B)** The replication timing profiles for all five cell lines displayed strong spatiotemporal structure, as measured by autocorrelation. **(C)** Pearson correlations among cell lines. These are within the expected range for different cell types, given previous studies [2,3].

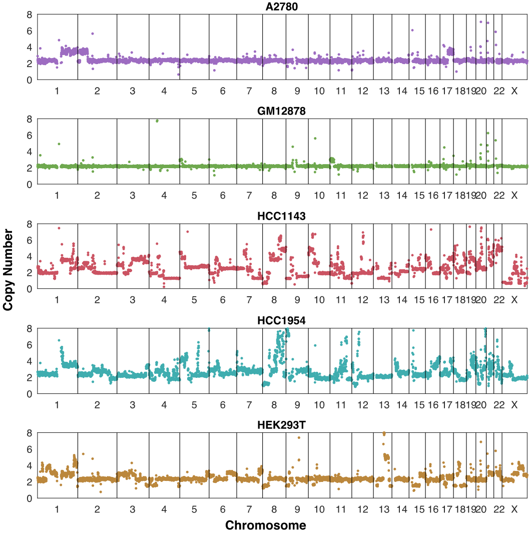

Figure S2. Copy number in 1Mb windows for the G1-phase fractions, following mappability- and GC-bias correction using GenomeSTRiP [4]**.** GM12878 is diploid and A2780 is near-diploid, while HCC1143, HCC1954, and HEK293T display many chromosome gains and losses. Normalization of the S-phase fractions against these G1-phase backgrounds was necessary to account for these changes in sequencing read depth that do not reflect DNA replication.

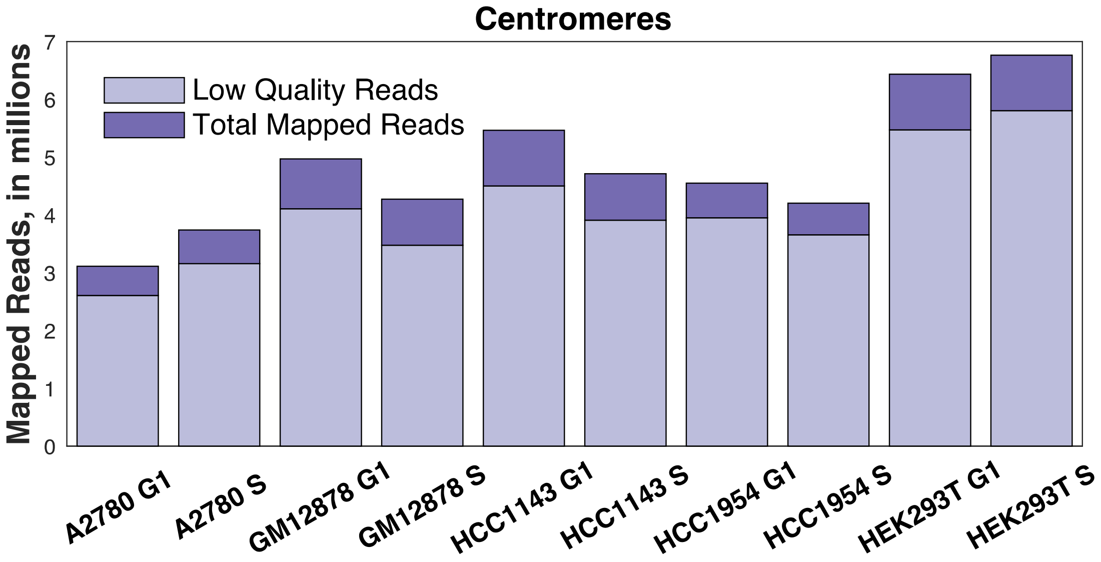

Figure S3. Approximately 85% of read pairs mapped to centromeres are flagged as low-quality and removed prior to analysis. This reflects the repetitive nature of the centromere reference sequences. Low-quality reads were defined as those reads with a BWA mapping quality (MAPQ) score < 10.

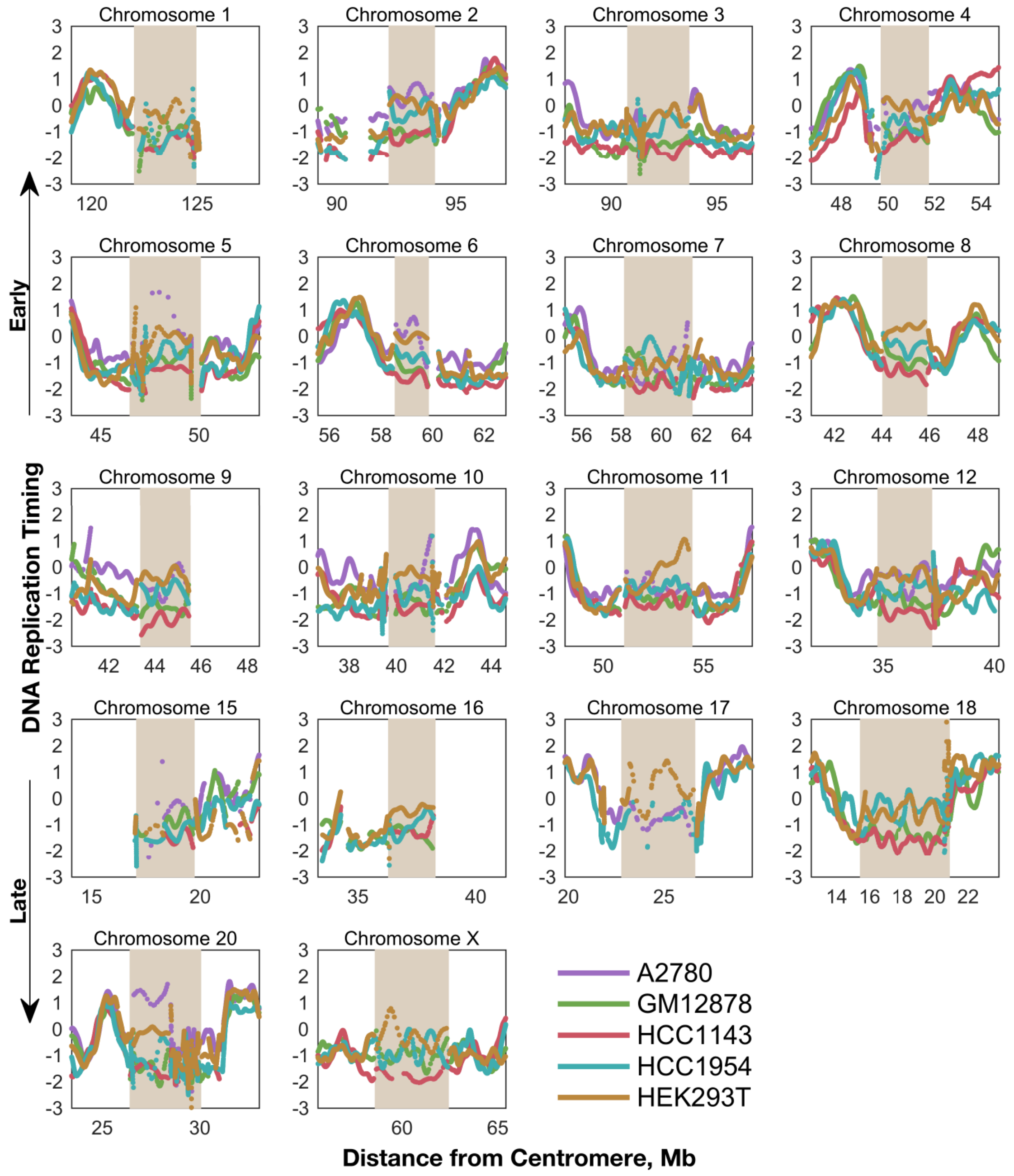

Figure S4. Cell lines display variation in centromeric replication timing across all chromosomes. Smoothed replication profiles for mappable (see **Figure 1**) centromeres in all five cell lines. HEK293T and A2780 tend to have earlier centromeric replication timing than the other cell lines.

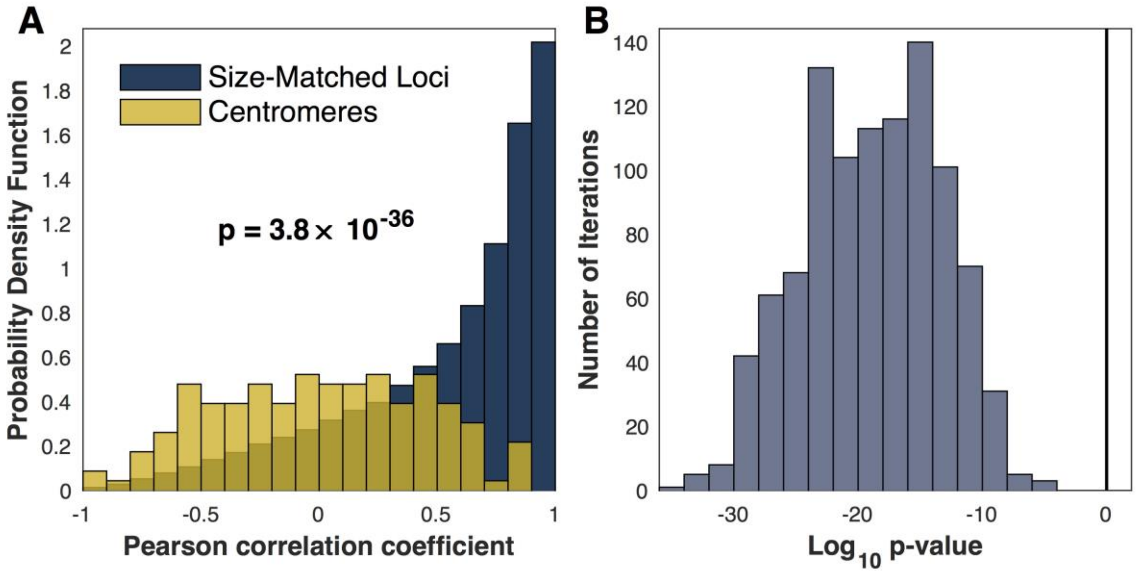

Figure S5. The broad distribution of pairwise correlations for centromeric regions is significantly different than expected by chance. (A) The distribution of pairwise correlation coefficients between cell lines for centromeric regions (gold bars) is significantly different from the distribution of pairwise correlation coefficients between cell lines for all possible size-matched windows (blue bars, two-sample Kolmogorov-Smirnov test, $p = 3.8 \times 10^{-36}$). **(B)** To account for the effects of sampling, we designated random size-matched genomic regions as the "centromeres" and calculated the correlation for these regions between cell lines, for 1,000 iterations. In 999 of 1,000 iterations, the distribution of correlation coefficients for an equivalent number of size-matched random loci was greater and significantly different from the observed distribution of centromeric correlation coefficients. Gray bars display the $\log_{10}$ p-value from a two-sample Kolmogorov-Smirnov test for each comparison (range: 10^{-34} – 10^{-4}). The black line indicates the Bonferroni-corrected p-value threshold of 5×10^{-5} for 1000 tests.

References

1. Koren, A.; Polak, P.; Nemesh, J.; Michaelson, J.J.; Sebat, J.; Sunyaev, S.R.; McCarroll, S.A. Differential relationship of DNA replication timing to different forms of human mutation and variation. *Am J Hum Genet* **2012**, *91*, 1033-1040, doi:10.1016/j.ajhg.2012.10.018.
2. Ryba, T.; Hiratani, I.; Lu, J.; Itoh, M.; Kulik, M.; Zhang, J.; Schulz, T.C.; Robins, A.J.; Dalton, S.; Gilbert, D.M. Evolutionarily conserved replication timing profiles predict long-range chromatin interactions and distinguish closely related cell types. *Genome Res* **2010**, *20*, 761-770, doi:10.1101/gr.099655.109.
3. Hansen, R.S.; Thomas, S.; Sandstrom, R.; Canfield, T.K.; Thurman, R.E.; Weaver, M.; Dorschner, M.O.; Gartler, S.M.; Stamatoyannopoulos, J.A. Sequencing newly replicated DNA reveals widespread plasticity in human replication timing. *Proc Natl Acad Sci U S A* **2010**, *107*, 139-144, doi:10.1073/pnas.0912402107.
4. Handsaker, R.E.; Van Doren, V.; Berman, J.R.; Genovese, G.; Kashin, S.; Boettger, L.M.; McCarroll, S.A. Large multiallelic copy number variations in humans. *Nat Genet* **2015**, *47*, 296-303, doi:10.1038/ng.3200.
